# Empowering Participatory Research in Urban Health: Wearable Biometric and Environmental Sensors for Activity Recognition

**DOI:** 10.3390/s23249890

**Published:** 2023-12-18

**Authors:** Rok Novak, Johanna Amalia Robinson, Tjaša Kanduč, Dimosthenis Sarigiannis, Sašo Džeroski, David Kocman

**Affiliations:** 1 Department of Environmental Sciences, Jožef Stefan Institute, 1000 Ljubljana, Slovenia; johanna.robinson@acs.si (J.A.R.); tjasa.kanduc@ijs.si (T.K.); david.kocman@ijs.si (D.K.); 2 Ecotechnologies Programme, Jožef Stefan International Postgraduate School, 1000 Ljubljana, Slovenia; saso.dzeroski@ijs.si; 3 Centre for Research and Development, Slovenian Institute for Adult Education, 1000 Ljubljana, Slovenia; 4 Environmental Engineering Laboratory, Department of Chemical Engineering, Aristotle University of Thessaloniki, 54124 Thessaloniki, Greece; denis@eng.auth.gr; 5 HERACLES Research Centre on the Exposome and Health, Centre for Interdisciplinary Research and Innovation, 57001 Thessaloniki, Greece; 6 Environmental Health Engineering, Department of Science, Technology and Society, University School of Advanced Study IUSS, 27100 Pavia, Italy; 7 Department of Knowledge Technologies, Jožef Stefan Institute, 1000 Ljubljana, Slovenia

**Keywords:** wearable sensors, particulate matter, activity recognition, machine learning, low-cost sensors, participatory research

## Abstract

Participatory exposure research, which tracks behaviour and assesses exposure to stressors like air pollution, traditionally relies on time-activity diaries. This study introduces a novel approach, employing machine learning (ML) to empower laypersons in human activity recognition (HAR), aiming to reduce dependence on manual recording by leveraging data from wearable sensors. Recognising complex activities such as smoking and cooking presents unique challenges due to specific environmental conditions. In this research, we combined wearable environment/ambient and wrist-worn activity/biometric sensors for complex activity recognition in an urban stressor exposure study, measuring parameters like particulate matter concentrations, temperature, and humidity. Two groups, Group H (88 individuals) and Group M (18 individuals), wore the devices and manually logged their activities hourly and minutely, respectively. Prioritising accessibility and inclusivity, we selected three classification algorithms: k-nearest neighbours (IBk), decision trees (J48), and random forests (RF), based on: (1) proven efficacy in existing literature, (2) understandability and transparency for laypersons, (3) availability on user-friendly platforms like WEKA, and (4) efficiency on basic devices such as office laptops or smartphones. Accuracy improved with finer temporal resolution and detailed activity categories. However, when compared to other published human activity recognition research, our accuracy rates, particularly for less complex activities, were not as competitive. Misclassifications were higher for vague activities (resting, playing), while well-defined activities (smoking, cooking, running) had few errors. Including environmental sensor data increased accuracy for all activities, especially playing, smoking, and running. Future work should consider exploring other explainable algorithms available on diverse tools and platforms. Our findings underscore ML’s potential in exposure studies, emphasising its adaptability and significance for laypersons while also highlighting areas for improvement.

## 1. Introduction

Exposure studies often rely on participants or subjects to provide information about their movements and activities relevant to the study. Time use diaries, or time activity diaries (TADs), have been extensively used to record specific activities and their relation to economic or health factors [1]. While TADs have been mostly paper-based, in the last decade, activity tracking has transitioned to smartphone apps and web-based applications, improving diary data quality [2]. On the other hand, there are indications that using smartphone apps can increase nonresponse levels due to several factors, e.g., not owning a smartphone and unfamiliarity with digital tools, though there are options available to overcome some of these issues [3].

Analysing the everyday activities of individuals can present a useful way to compartmentalise human behaviour and subsequently assess exposure to stressors, such as pollution or noise. Strong evidence exists that different activities increase exposure to stressors, e.g., elevated levels of airborne particulate matter when dusting, folding clothes, making a bed [4], smoking cigarettes [5], vaping [6], or walking/vacuuming on carpeted flooring [7,8], and increased exposure to noise on public transport [9]. Manually recording activities by a large group of individuals can be imprecise or require more resources [10]. An important constraint is temporal resolution, which has to be suited to participants/subjects’ availability and responsiveness. When individuals self-report activities, there is little control over how precise the reports are, especially when taking into account recall bias and reliability [11,12,13]. In this context, participatory research, a collaborative approach that involves stakeholders, particularly those affected by the issue being studied, in all aspects of the research process, from defining the problem to collecting and analysing data, may offer a more integrated and accurate method of data collection. Reviewing TAD data in this study, in collaboration with the participants, showed a possible error rate of up to 5–10% for each activity. To reduce the probability of human error, different approaches are employed, e.g., user-cantered study design to construct better TADs [14] and using GPS and other variables as activity identifiers to reduce manual input [15,16,17].

Different classification algorithms developed over the past decades could potentially classify different activities by using data recorded with sensors as learning data. Equipping each individual with low-cost sensors would provide data about their movement, physiology, and environment. Relying solely on movement data or environmental data does not necessarily provide enough information to predict complex activities. This study utilised machine learning methods for classification, in combination with sensor and activity data, to provide a proof of concept for an alternative to manually recording complex activities. Furthermore, the approach was centred on analysing the usefulness of these tools to non-expert users involved in participatory research. To this end, two groups of participants, equipped with biometric and environmental sensors, recorded their activities with different temporal resolutions. The collected data were used to learn three different classification algorithms, observe how accurately each of them classifies simple and complex activities, and determine the role of different temporal resolutions. Overall, the aims of this study are:

(i) To evaluate the effectiveness of using a combined dataset from environmental and biometric sensors for the recognition of complex individual activities, utilizing classifiers selected for their transparency, interpretability, and accessibility to laypersons. This involves comparing the predictive performance of different classifiers to ascertain their suitability in a participatory-based urban health stressor context.
(ii) To investigate how different temporal resolutions of the collected data influence the predictive performance of each classifier, thereby determining the optimal data granularity for accurate activity recognition in urban health studies.
(iii) To assess the individual contributions and overall value of the environmental and biometric sensors used in the study, particularly focusing on their role in enhancing the accuracy of human activity recognition for complex urban activities.
(iv) To assess the role of these classifiers and sensors in empowering lay individuals for participatory urban health research. This involves enhancing the accessibility and understandability of human activity recognition technology, thereby enabling more effective community involvement in urban environmental health studies.

### 1.1. Air Quality and Environmental Data from Personal Monitors

Low-cost personal sensors and monitors that measure ambient conditions are becoming increasingly popular. Particulate matter concentrations, temperature, relative humidity, and various gases are just a few of the parameters that can be measured with low-cost personal sensors and monitors. These devices have certain drawbacks, mainly the uncertainty of their results [18,19], although they have been improving in the past years [20] and are “cautiously encouraged” for monitoring indoor air quality [21].

Certain complex activities, e.g., cooking, cleaning, and smoking, are characterised by distinct environmental conditions (smoke, resuspension of particles, high humidity), which could potentially provide enough data for a classification algorithm to identify them. Environmental sensors for temperature, humidity, and light have been successfully used to aid in activity recognition [22], including using humidity, CO_2_, and temperature to classify specific (simple) activities in a single room [23]. To the best of our knowledge, individual-level particulate matter concentration, in combination with biometric data, has not been used for HAR.

### 1.2. Human Activity Recognition

Human activity recognition (HAR) methods have notably evolved, enabling the distinction of various activities using low-cost sensors without manual input. Recent advancements have shown the potential of HAR in applications such as mobile healthcare, smart homes, and fitness monitoring [24]. While earlier research focused on utilising sensors like accelerometers, compasses, gyroscopes, barometers, magnetometers, and GPS present in smartphones to predict specific actions, events, or activities such as walking, running, and falling [25,26], dedicated activity trackers (usually worn on the wrist) are more sophisticated and provide more accurate data [27,28]. On the other hand, even as these devices are better at HAR, they still have a high error rate (mean absolute percentage error (MAPE) of >50%) for more complex activities (dusting, cleaning, playing cards, etc.), but provide heart rate measurements with lower mean errors, i.e., between −3.57 bpm and 4.21 bpm, and MAPE of up to 16% [29,30]. In some instances, they have shown accuracy >90% for certain complex activities, e.g., smoking [31], by utilising hand gestures. Sensors measuring ambient conditions have been deployed in various HAR-oriented research projects.

#### 1.2.1. HAR Challenges and HAR Pipeline

Several technical challenges persist in HAR, according to Chen et al. [32]:

1. Difficult feature extraction is due to activities having similar characteristics.
2. The high cost and time-intensive nature of activity data collection leads to annotation scarcity.
3. Person-dependent activity patterns, temporal variability of activity concepts, and diverse sensor layouts in individuals result in sensory data heterogeneity.
4. Composite or complex activities encompass several actions, making them more difficult to classify. Concurrent and multi-occupant activities, where an individual performs multiple activities simultaneously or with multiple people, add to the complexity.
5. A high computational cost is associated with HAR systems that have to provide instant responses and fit into portable devices.
6. The privacy and interpretability of the collected data have to be considered.

A recent HAR research pipeline has been introduced, offering a structured approach to human activity recognition [33]. This pipeline consists of nine interconnected components, guiding research from equipment selection to real-world application. It emphasises the importance of data acquisition, segmentation, annotation, signal processing, and feature extraction. The final phase integrates the research into practical scenarios [33]. In this research, the proposed pipeline has been implemented into the workflow. The equipment used was chosen for its relevance in capturing data across a range of activities in variable environmental conditions in Ljubljana, Slovenia. The data acquisition phase was focused on quality data capture amidst environmental variations. The subsequent segmentation and annotation phases were critical for accurately categorising diverse activities from indoor to outdoor settings. In signal processing, we processed the data to filter out noise. These methodologies were applied in the scope of this work, with an emphasis on real-world applicability and data interpretation. Each stage addressed specific challenges, including data quality and environmental adaptability.

#### 1.2.2. Deep Learning Models for HAR

Deep learning models, such as those based on the self-supervised learning framework SimCLR, have showcased competitive performance in HAR using ambient sensor data [34]. In smart homes, the use of ambient sensors has become crucial due to the increasing demand for applications that can recognise activities in real-time [35]. Transformer-based filtering networks combined with LSTM-based early classifiers have been proposed to address the challenges posed by unrefined data in real-time HAR [35]. Cross-house human activity recognition is another area of interest, aiming to use labelled data from available houses (source domains) to train recognition models for unlabelled houses (target domains) [36]. Wearable devices have also played a pivotal role in HAR, supporting the detection of human activities without the need for environmental sensors like cameras [37]. These devices offer the advantage of not constraining users to remain in controlled environments. A deep understanding of the situations in which activities are performed is essential for applications in domains like safety, security, surveillance, and health monitoring [37].

#### 1.2.3. Classification and Shallow Algorithms for HAR

While deep learning has shown high accuracy, usefulness, and advancements in HAR [38], shallow machine learning algorithms continue to demonstrate effectiveness in this field. Classification specifically is one of the major tasks in machine learning, where an algorithm learns, from examples in the training data, how to assign a specific class to the testing data. A task of this kind is to classify emails as “spam” or “not spam”. In its most rudimentary form, an algorithm would check in a labelled training dataset which words or phrases are associated with a spam email and which are not. With these learned associations, the algorithm would be used on a new (testing) dataset, classifying new emails into “spam” and “not-spam”. This is an illustrative task of binary classification, whereas activity classification usually requires multi-class classification, where there are several different classes, such as walking, running, cleaning, smoking, and so on. A variety of algorithms can be used for classification, including *k*NN [39], decision trees [40], Naïve Bayes [41], random forests [42], gradient boosting [43], support vector machines [44], etc.

Classifiers have been used in various HAR applications that use smartphones and low-cost activity trackers or other mobility sensors, in some cases with accuracy >98% [45], and in most cases >80% [46,47]. Combining these data points with ambient conditions, such as temperature and relative humidity measured with a smartphone (which has certain drawbacks [48]) or with static sensors, has shown up to 99.96% of correctly classified activities, such as walking, sitting, cycling, running, and other similar, less complex activities [49,50]. Specifically, decision tree classifiers (DTC), random forest classifiers (RFC), and K-nearest neighbours (KNN) have been utilised to recognise activities such as walking upstairs, walking downstairs, and walking normally, among others. The RFC model exhibited superior performance, achieving an accuracy score of 97.67% [51]. A proposed framework for HAR using smartphone sensors employed random forest, decision tree, and k-nearest neighbour classifiers, achieving an accuracy of 93.10% [52].

These approaches have utilised an array of different classifiers with varying results, where some algorithms, such as Naïve Bayes, achieve an average accuracy of 43.29% as compared to random forests, with the accuracy of 99.96% [50] and 99.86% [49]. Fewer studies have attempted to identify more complex activities, such as cooking, cleaning, gardening, playing, smoking, and others, as they are more difficult to characterise or distinguish from each other. Dernbach et al. [53] report over 90% accuracy for simpler activities for all classifiers (except Naïve Bayes with ~74%), while the accuracy for more complex activities was ~50% (only for K-star, otherwise between 35% and 50%). As complex and simple activities are broad terms, it is useful to define them. Sousa Lima et al. [54] provide a good explanation to delimit these two types of activities: “Simple or low-level activities are those activities that can only be recognized by analysing data from one or more sensors in a short period of time (e.g., walking and running). While complex or high-level activities can be seen as a set of low-level activities that can be recognized over a long period of time (e.g., work and shopping).” In this study, the scope of definitions is broadened to encompass various sensor types essential for HAR, including movement, biometric, and environmental sensors. Simple activities such as running, sleeping, resting, and sports typically require only a single device for effective monitoring. Conversely, complex activities like cleaning, cooking, playing, and smoking often demand additional data on ambient conditions. The research primarily investigates complex activities while also considering some simpler ones.

To promote the wider adoption of machine learning and classification methods, particularly in participatory and citizen science initiatives where individuals actively contribute data and collaborate in research, it is preferential to prioritize the explainability and accessibility of the algorithms and tools employed. In this context, WEKA, a collection of visualisation tools and algorithms designed for data analysis and predictive modelling, is identified as a promising tool [55]. Combining data from different environmental and biometric sensors and devices could provide enough information to distinguish different complex activities and somewhat resolve the listed challenges. Employing explainable algorithms available in accessible and user-friendly environments could lead to a wider adoption of machine learning and classification for HAR in participatory-based research.

## 2. Methodology

### 2.1. Data Collection

Two sets of data were used for this research, collected from participants living in Ljubljana, Slovenia:

The first set (group H—hourly data) was collected as part of the ICARUS H2020 project from 88 participants [56]. The participants were involved in the winter (February to March 2019) and summer (April to June 2019) seasons of the campaign for approximately 7 days and were equipped with two sensor devices: a smart activity tracker (SAT) and a portable particulate matter (PM) measuring device (PPM). Basic personal information was obtained from each participant (age, body mass, sex, etc.). All participants had to fill out a TAD, where information about their activities was provided for each hour. They could select their hourly activity from 7 indoor activities (resting, sleep, playing, sports, cooking, smoking, cleaning) and 2 outdoor activities (running, sports). The activities chosen to be included in the TAD were based on the criteria developed within the ICARUS project, based on available research and activity pattern databases [57]. Sensor data, collected with a 1-min resolution, were aggregated to a 1-h resolution by calculating the mean value. A detailed description of the sampling campaigns was published by Robinson [58].

The second set (group M—minute data) was collected from September to November of 2020 from 18 participants. They were equipped with the same devices as the first group. An important distinction was that they (a) had more activities to choose from and (b) had to log activity data on the scale of minutes, not hours. The activities used for group M were modified activities from the initial ICARUS TAD. Sensor data with a 1-min resolution were used as-is.

All participants involved in the study provided their informed consent. Ethical approval for the ICARUS project in Slovenia was obtained from the National Medical Ethics Committee of the Republic of Slovenia (approval nr. 0120-388/2018/6 on 22 August 2018). The data in this paper were selected only from participants in Slovenia, and all methods were performed in accordance with the relevant guidelines and regulations.

**Figure 1:**
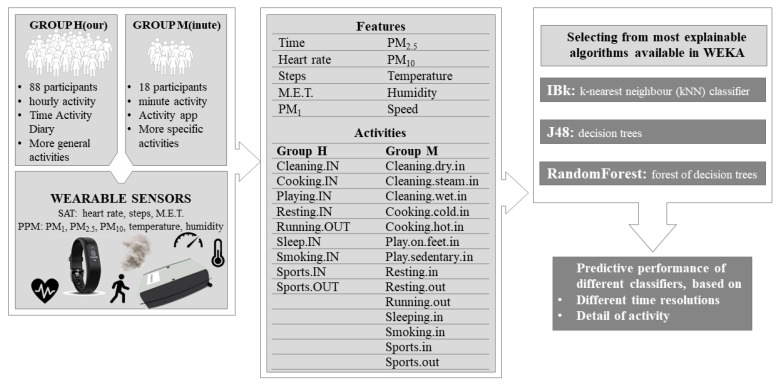
Schematic representation of the overall methodology and data flows used in this work.

A graphic representation of the methodology and dataflows used in this work is shown in Figure 1.

#### 2.1.1. Smart Activity Tracker

A Garmin (Garmin, Olathe, KS, USA) Vivosmart 3 activity tracker was strapped to each participant’s wrist for the entire duration of the data collection period, except for two hours when the device had to be recharged. Information about the participant (sex, age, body mass, height, etc.) was logged into the device before deployment. The temporal resolution of the data was one minute. Data for average minute heart rate [beats per minute], steps [number of steps], and metabolic equivalent of task (M.E.T.) [between 0.01 and 45.60] was collected from each participant. The SAT provided several other variables, though they were not relevant to the scope of this research. Raw data, e.g., accelerometer, was not accessible.

The device measured heart rate using photoplethysmography (PPG) [59], with the wrist as the preferred location for its cost-effectiveness and convenience. According to information disclosed by Garmin, this specific device contains four sensors: a Garmin Elevate^TM^ write heat rate monitor, a barometric altimeter, an accelerometer, and an ambient light sensor [60]. Validation studies confirm the Garmin Vivosmart’s accuracy in capturing relevant data, including in older adults [61,62,63], though caution is advised for energy expenditure and high-intensity activities [64,65].

#### 2.1.2. Portable Particulate Matter Sensing Device

This low-cost PPM device was developed for the ICARUS project by IoTECH (IoTECH Telecommunications, Thessaloniki, Greece) using a Plantower pms5003 sensor (Nanchang Panteng Technology Co., Ltd., Nanchang, China). This sensor uses the optical particle counting principle to measure particle size and mass concentration in real time. A fan draws particles into a beam of light, illuminating each particle as it passes through, with scattered light being recorded on a photodetector and converted into an electrical signal. The device provided data at a one-minute resolution. Participants carried it with them the entire period, strapped to their clothes, handbags, backpacks, or something similar. When sedentary, they were instructed to “have it in the same room, as close as possible”, as the device needed to be recharged every six to seven hours or continuously plugged into a power source. The pms5003 sensor consistently demonstrates accuracy in both short-term and long-term evaluations, exhibiting moderate to high correlation with reference instruments in various settings [66,67,68]. While some studies suggest minimal drift over time [68,69], others recommend regular calibration, especially in high-humidity environments [67]. Additionally, the Plantower sensor recorded ambient temperature and humidity, and the ancillary GPS component provided data on speed of movement. Group M was provided with a power bank with a 10.000 mAh capacity, which prolonged the use of the device to ~24 h. The PPM provided minute-resolution data for PM_1_, PM_2.5_, PM_10_, temperature [°C], relative humidity [%], and speed [km/h]. Some other variables were also provided by the PPM, though they were out of the scope of this research. The PPM was validated by co-location with reference research-grade sensors [70].

#### 2.1.3. Activity Recording

Group H was provided with 7 blank daily time activity diaries (TADs), where they were able to fill in circles for each activity they did for every hour of the day. These files were collected and digitalized. Information about all indoor and outdoor activities was used. An example of a TAD can be found as supplemental information in Novak et al. [71].

Group M installed the Clockify app [72] on their smartphone, which had activities already pre-set by the research team on the online portal. Several activity-tracking apps were tested and reviewed, and though the Clockify app was generally meant as a time-tracking app for work and projects, it had the functionalities that were needed for this research. Each participant selected the activity they were beginning to perform, and the timer would start. After they finished the activity, they would select the next activity, which would automatically finish the first one. The time stamps had date, hour, minute, and second information. While the activity data technically had a 1-s resolution, it was rounded to the nearest minute. The reasoning was fourfold: (1) a few instances of activities with a duration of <1 min, (2) the compiled dataset would be unnecessarily large, (3) the changes between activities included in the analysis are not relevant at <1-min resolution, and (4) all sensor data had a 1-min resolution. The recorded data were exported from the Clockify portal in csv format.

Recording more than one activity simultaneously was deemed out of the scope of this research. In the case of Group M, recording more than one activity at once was not possible in the app. On the other hand, there were some instances of participants recording more than one activity per hour in Group M. Generally, these were not the same variables as those used in this research. In the few cases where this overlap happened, the first activity from the right side of the TAD was selected.

All annotations of data and any segmentation were based on the activity data provided by the participants.

### 2.2. Dataset Overview

Sensor, TAD, and Clockify portal data were harmonised and compiled into two datasets: group H with 228,267 instances (per-minute recordings of all variables), and group M with 70,139 instances. Each instance was associated with 10 variables (time, PM_1_, PM_2.5_, PM_10_, temperature, humidity, speed, heart rate, steps, M.E.T., activity):

- time—indicating a time of day or a specific hour when the measurement took place (from 0 to 23)
- PM_1_, PM_2.5_, and PM_10_—particulate matter concentrations in three size classes, recorded as non-negative integer values (PPM)
- temperature, humidity—ambient temperature and humidity, recorded as a float value (PPM)
- speed—calculated based on GPS module data, recorded as a float value (PPM).
- heart rate—heart rate per minute, recorded as a positive integer value (SAT).
- steps—number of steps per minute, recoded as a non-negative integer value (SAT).
- M.E.T.—a non-negative integer value (SAT).
- activity—recorded on TAD or in the Clockify app.

The minimum requirement for each instance to be included in the dataset was an activity label and at least one non-empty variable. Data preparation was done in R [73].

The number of included instances of each activity for each dataset (group M and group H) is listed in Table 1. All activities were capped at 5000 instances for each group. When >5000 instances were available, a random selection was made from the dataset. Resting and sleeping were capped for group M. The ceiling was determined based on several iterations, which showed a considerably longer time to build the models without having an impact on the overall performance.

Basic statistics for all numeric variables in the final datasets are presented in Table 2. All values were within expected limits. All PM variables had a ceiling fixed at 180 µg/m^3^ as the highest possible value; otherwise, the mean, median, and quartile values are as expected. The mean and median values for speed are low, as >20 km/h values were removed, as there are no activities included in this research where speed could be >20 km/h. PM statistics are similar between the groups. There are some differences in max and min temperature and relative humidity, which is due to a larger and more diverse dataset for group H and data from two seasons. Speed, heart rate, steps, and M.E.T. do not show wide discrepancies. As individuals, on average, spend most of their time in a sedentary or stationary position, the low median, mean, and quartile values reflect this. Similarly, the highest values reflect vigorous movement, e.g., running, producing >200 steps per minute, and an M.E.T. of 15. These results show that the two datasets are quite similar when observed through basic statistics, which was a key aim that was set when collecting data for group M. The two groups should have the same general characteristics and differ only in the temporal resolution of the data collected to facilitate an accurate comparison of the classification results. The mean values of all variables for each activity are available in the Supplementary Information. These show that the values are in line with expectations as there are generally higher concentrations of PM outdoors than indoors, though this is somewhat dependent on season, time of day, and specific activity [74,75].

### 2.3. Classifiers Used

In this research, shallow algorithms were opted for over deep learning techniques, given their inherent advantages tailored to the objectives of this research. While deep learning methods frequently offer high accuracy, they come with increased computational demands. This becomes a challenge when classifications are executed on devices with limited computational power, such as smartphones, wearable sensors, or standard office laptops. Although these demands can be mitigated through sophisticated feature extraction methods [76], it might render the approach less intuitive for those not well-versed in machine learning. Importantly, certain shallow algorithms are recognised for their transparency and interpretability. For instance, tree-based algorithms, like decision trees and random forests, are visually representable, making them more comprehensible to laypersons [77]. The k-nearest neighbours (kNN) algorithm, with its principle of similarity, is also straightforward in its logic. In this study, shallow algorithms not only facilitate easier explanations for research participants but also ensure that the data analysis remains accessible to researchers. As artificial intelligence becomes increasingly integrated into research and policy making, the emphasis on the explainability of these algorithms grows [78,79]. Notably, the trade-off between model accuracy and interpretability in AI has been a focal point in recent research, with a survey paper offering an in-depth analysis of explainable AI methodologies and suggesting future research avenues to optimize this balance [80].

The classifiers chosen for the tasks outlined in this research were selected based on the requirements outlined in Section 1 and Section 2.3. These requirements can be summarised into four criteria:

1. All selected algorithms must be appropriate for this task, based on their use in existing literature and proof-of-concept cases, and show promising results in terms of accuracy in published research.
2. They should be considered (easily) explainable to laypersons, with the processes used being transparent and understandable.
3. Accessibility must be considered, i.e., the algorithms must be available in a user-friendly, GUI-based experimental environment allowing access to laypersons, e.g., WEKA.
4. The algorithms used should be executable on devices with limited computational power, such as smartphones or office laptops, proving results in a reasonable time frame, as per the aims of specific research.

Based on the outlined criteria, the following classifiers were chosen: *k*NN, decision trees, and random forests. All analyses were performed using the WEKA 3.8.3 [55] “Explorer” application. The specific classifiers within WEKA that were used in this research are listed in Table 3, which also contains short descriptions of each of the classifiers.

### 2.4. Parameter Settings for the Classifiers

The settings for all classifiers were at their WEKA defaults. IBk used 1 nearest neighbour for classification and did not perform distance weighting. J48 trees were pruned. The RF contained 100 trees.

### 2.5. Feature Ranking Using the Relief Approach

Not all attributes in the dataset are necessarily useful for classification models, and some can be omitted. In turn, this can reduce the time and computational cost of building the model. The features in these datasets were ranked using the Relief approach, i.e., the Relief Attribute Evaluator in WEKA, with 10-fold cross-validation. Relief “evaluates the worth of an attribute by repeatedly sampling an instance and considering the value of the given attribute for the nearest instance of the same and different class” [82].

### 2.6. Performance Metrics

There are many measures of the performance of classifiers, typically defined for each class value (and then averaged across the different class values). These include true positive and false positive rates, precision, recall, F-measure, and others. Classification accuracy is defined as the percentage of instances that have been classified correctly. It is the most commonly used indicator of performance. Another performance metric is Kappa (K), which allows for direct comparison between models as it shows how closely the classified instances match the labelled data while also considering random chance (agreement with a random classifier).

Performance metrics are typically calculated based on the entries of the confusion matrix for a given classifier C and a given dataset D. The entry in row x and column y specifies the number of instances from D that actually belong to class x but have been classified as class y by the classifier C. The diagonal entries of the matrix specify the numbers of correctly classified instances. The confusion matrices for all three classifiers and the two groups are provided in the supplementary information.

For a given class x, the diagonal entry corresponds to the number of true positives (TP) for class x. The sum of all non-diagonal entries in column x corresponds to the number of all instances incorrectly classified as x (false positives). Based on TP and FP, the precision for x is calculated as:

$${Precision}_{x}=\frac{TP}{TP+FP}$$

Recall (sensitivity) for class x is defined as the ratio of true positives to the total number of instances that truly belong to class x:

$${Recall}_{x}=\frac{TP}{TP+FN}$$

The F-measure is a metric that combines precision and recall into a single score, providing a balanced measure of a classifier’s performance:

$$F_{x}=\frac{2*{Precision}_{x}*{Recall}_{x}}{{Precision}_{x}+{Recall}_{x}}$$

A receiver operating characteristic curve, or ROC curve, is a graphical plot that illustrates the diagnostic ability of a probabilistic binary classifier, and the area under the ROC curve (ROC-AUC) is also often used as a performance metric. The ROC curve plots the true positive rate (TPR), also known as sensitivity or recall, against the false positive rate (FPR) at various threshold settings. FPR is calculated as:

$$FPR=\frac{FP}{FP+TN}$$

These metrics and the ROC curve provide valuable insights into the performance of classifiers by considering both the ability to correctly classify instances (precision, recall) and the ability to evaluate a model’s performance across different decision thresholds (ROC curve).”

The performance of all three classifiers on unseen cases was estimated by using the 10-fold cross-validation procedure. Cross-validation reduces the variance in the performance estimates by averaging over different partitions of the dataset. The dataset is divided into 10-subsets (folds), which in turn are used as testing sets, while all remaining instances are used as training instances. This procedure ensures that every instance from the dataset appears in the test set exactly once.

## 3. Results and Discussion

### 3.1. Feature Importance and Ranking

In the analysis of feature importance and ranking, the focus was on identifying significant features influencing activity recognition in groups H and M. This was necessary for understanding activity patterns in relation to environmental exposures.

Time (hour of day) was the top-ranked feature for both groups, aligning with the expectation that certain activities are time-specific. Heart rate, humidity, and temperature were also important, with their order of importance varying between groups.

Notable differences between the groups were observed beyond the top four features. For group M, Table 4 indicates that PM_10_ and PM_2.5_ were ranked 5th and 6th, with PM1 at an average rank of 9.2. This variation may be due to different temporal resolutions in recording sensor and activity data. In group H, as shown in Table 5, steps, speed, and metabolic equivalent of task (M.E.T.) ranked higher, followed by particulate matter (PM) variables with the lowest average merit.

Due to the small dataset size, no attributes were excluded. Therefore, models were trained using a comprehensive set of features: PM_1_, PM_2.5_, PM_10_, humidity, temperature, speed, heart rate, steps, M.E.T., and time.

The analysis showed expected patterns, such as the high ranking of time, and less intuitive differences between the groups in the ranking of PM variables and physical activity indicators. These findings emphasise the complexity of human activity patterns in relation to environmental exposures and the need to consider a range of features in such studies.

### 3.2. Overall Predictive Performance of Classifiers

Table 6 shows a comparison of the most relevant metrics for all the classifiers used in this research for groups H and M. Random Forest (RF) shows the highest correctly classified (CC) values for both groups, and IBk shows the lowest CC values. For group H, the percent of correctly classified instances increases gradually from IBk to J48 (Δ11.3%) to RF (Δ6.4%). On the other hand, for group M, the share of correctly classified instances jumps up from IBk to J48 (Δ42.6%), but then only marginally increases from J48 to RF (Δ0.3%). This difference could be a consequence of the different number of instances for each activity between the two groups. Some activities in group M have <500 instances, while all activities in group H have 5000 instances. The trend for Kappa is similar to CC, though all values for J48 and RF are ~0.06 lower than the correctly classified percent (divided by 100). Though there is some disagreement on the applicability of the Kappa statistic in the context of “The Paradox of Cohen’s Kappa” [83,84] and what the guidelines are for evaluating it [85], in this context, a value of >0.7 can be interpreted as moderate to strong agreement. Both J48 and RF fall in this category for group M. For group H, all Kappa values are <0.5. Importantly, the difference between J48 and RF for group M is only 0.01.

Calculated values for TP and precision again follow the CC and Kappa metrics, showing that J48 and RF provide the highest precision. The RF model for group M has the highest ROC-AUC (0.97), indicating the lowest FP and highest TP rate.

An important evaluator that is not included in the table is the time it took to construct each model. For IBk, the time to build the model was <1 s for both groups. This is a positive aspect for IBk, though all the evaluation metrics show that this model is not suited for this type of data in comparison with J48 and RF. For group H, J48 took 4.67 s to build the model and 0.42 s for group M. In contrast, the RF-based model took 121.89 s for group H (more than 57 times as much time as J48) and 24.16 s for group M (26 times as much time as J48). As these two models perform very similarly based on the evaluation metrics, the time it takes to build and cross-validate the model is a relevant factor when considering which one to use. In the case of the group M subset of data, it would be efficient to use the J48 classifier, as the improvement in the correctly classified percent of instances does not offset the time and processing power that have to be allotted. Real-world applications, based on collecting data with personal sensors, experience larger volumes of instances and would have to account for considerably longer run times. When a ML approach is applied to improve the classification of activities and reduce the probability of human errors, time and processing power should be considered. In line with the 5th challenge listed in Section 1.2, reducing the number of unnecessary instances, e.g., for sleeping, and selecting a more fit-for-purpose algorithm would reduce the computational cost associated with activity recognition.

### 3.3. Predictive Performance Per Group and Activity

Results comparing the predictive performance of the used classifiers for group H (Table 7) show similar results as described in Section 3.2. J48 and RF show an overall higher TP rate, precision, recall, F-measure, and ROC-AUC values compared to IBk. These differences are more obvious in simpler activities, i.e., running, sleeping, and sports, with a 0.3 difference in ROC-AUC between IBk and J48/RF and a ≤0.2 ROC-AUC difference for other, more complex activities. This result highlights the drawbacks of hourly recorded activity data, with less resolution on dynamic changes in more complex activities.

The IBk results for group M showed several activities with values of 0 in all evaluation classes, having a considerably worse predictive performance result than J48 and RF classifiers, as evident in Table 8. Running stands out in the ROC-AUC metric for IBk, with a relatively low FP rate compared to its TP rate. Unique characteristics of running, compared to other activities, such as high values for heart rate, speed, steps, intensity, and lower temperatures and PM concentrations, could contribute to better predictive performance. J48 and RF show a ROC-AUC and FP value of 1 and 0, respectively, for running. Results for group M show similar patterns as group H, with simpler activities showing better performance for all classifiers. On the other hand, group M TP and precision values are on average higher compared to group H. Cooking has a TP rate of 0.5 in group H, and while the TP rate for cooking.cold.in shows a value of 0.4, cooking.hot.in had a TP value of 0.8. The latter is associated with specific environmental conditions that can make it more distinguishable from other activities, e.g., higher temperatures and PM concentrations. Contrary to this assumption, the activity of playing does not show the same pattern. Playing.on.feet, associated with dust resuspension and an elevated heart rate, would be more distinctive than playing.sedentary. Although, on average, group M playing activities have better metrics compared to group H.

These conclusions are corroborated by the confusion matrix results available in the SI. Activities with value definitions have more misclassified instances, in contrast to well-defined activities. For example, out of the 5000 instances of resting in group M for J48 (Table S4 in Supplementary Material), 3738 are correctly classified. Out of the incorrectly classified, two-thirds (818) are labelled as sleeping and one-fifth as cooking.hot.in. Moreover, activities often have a high number of their misclassified instances labelled as resting. Out of 769 instances of smoking, 102 are misclassified as resting. On the other hand, 96 instances are misclassified as cooking.hot.in, which would be expected as both activities can show high concentrations of PM.

Furthermore, sleeping and resting have well-defined time intervals, a low heart rate, and no movement. They are consistently indicated by all participants and evenly distributed. Unlike other activities, sleep is uninterrupted for several consecutive hours, resulting in minimally distorted minute values within an hour. For instance, if a person runs for only 20 min but claims it is the main activity for that hour, only 1/3 of the data support this claim, while the remaining 40 min include other activities.

Resting is also somewhat characterised by longer, consecutive time intervals without interruptions. It also has a high FP rate and low precision in both groups. It is the second most frequent activity chosen by participants (behind sleeping) in the study, frequently overlapping with other activities. Resting could be understood as a “default” activity, chosen when no other activity fits the description. It is a vaguely defined activity and open to interpretation. Participants tend to include various activities under this term, e.g., reading a book, playing board or computer games, watching television, chatting with friends, taking a leisurely walk, napping, having a dinner party, etc. All of these activities can differ in many aspects, such as heart rate, movement, speed, or PM concentrations, which would make accurate predictions more difficult. While more detailed activity classification would improve on this point, it would increase the burden on participants.

### 3.4. The Added Value of the Devices Used

One aim of this research was to determine the respective contributions of the two devices used—the SAT and PPM—to the performance of activity classification. An assessment was conducted in WEKA based on the data collected in Group M, as it showed the best performance between the two groups. The RF and J48 classifiers were used to classify the data from Group M (1) without the data collected with the PPM, i.e., all PM data, temperature, relative humidity, and speed (no PPM), (2) without just the particulate matter data (no PM), and (3) without the data collected with the SAT, i.e., heart rate, M.E.T., and steps (no SAT). The results showed, as evident in Table 9, that in the case of J48, the share of correctly classified instances is not reduced much if the PM or SAT data were removed (by 0.7% and 1.4%, respectively). On the other hand, if the PPM part of the dataset is removed entirely, the share of correctly classified instances falls to 60.1%. The RF models show similar values and trends.

However, the overall number does not show certain nuances, e.g., the results without the SAT data show a lowered TP rate for sports.out from 0.8 to 0.6 (for J48). Though the difference is not as evident for running and sports.in, there is still a small decline in classification accuracy. This result shows that for specific activities, the SAT data increase accuracy. Similarly, for the dataset without the PM data, accuracy for smoking, running outside, and playing is lower, all of which are activities with increased exposure to PM, though the difference is less pronounced than with the absence of SAT data. On the other hand, removing the entire PPM dataset (for RF) shows worse accuracy for all activities, especially for playing.on.feet (−0.4), smoking (−0.3), play.sedentary (−0.3), and cleaning.wet (−0.2). The latter could be explained by the absence of data on relative humidity.

Collecting data on PM concentrations and other environmental variables is a partial improvement on the 1st and 4th technical challenges listed in Section 1.2. With more specific data on environmental conditions, the characteristics are more distinct and can improve feature extraction. Moreover, as complex activities have several actions associated with them, more data on the overall environment could offset the lack of data on these specific activities.

## 4. Conclusions

### 4.1. Summary of Results

Two groups of participants were equipped with devices that measured their exposure to PM and their physical activity, while they logged their activity data with a paper TAD with hourly resolution (group H) and a smartphone app with minute resolution (group M). The primary aims were to evaluate the effectiveness of combining low-cost personal environmental and biometric sensor data for recognising individual activities and to assess the impact of data temporal resolution on the performance of different classifiers. These classifiers were selected for their proven efficacy, understandability, and accessibility, aligning with our objective of empowering lay individuals with HAR technology in participatory urban health studies. Successfully achieving these aims could significantly reduce or eliminate the need for manual activity recording in exposure studies, paving the way for a broader application of machine learning and classification methods in HAR, particularly in participatory-based research settings.

Results showed improved accuracy when (1) the activity time resolution was changed from 1-h to 1-min resolution and (2) more vague activities, e.g., cleaning, cooking, and playing, were divided into more detailed categories. Most misclassified instances belong to activities with vague definitions (resting, playing), while well-defined activities (smoking, cooking, cleaning, running) have fewer misclassified instances. Accuracy increased for all activities, especially playing, smoking, and running, when the environmental sensor data were included.

All the used classifiers for group H showed accuracy above 35%, with RF being the most accurate with 52.9%. As the training data consisted of hourly labelled activities, this meant lower resolution and more errors (some activities do not last an hour, and most do not last exactly a set number of full hours). An improvement in labelling data by the minute was proposed and evaluated with group M, which showed a noticeable improvement in all measures of performance (e.g., the accuracy of ~77% for J48 and RF models). This was an expected outcome, as the sensor data were also recorded in minute intervals and provided a good starting point to achieve activity prediction without resorting to manually recording data, which are prone to errors.

All of the models, for both groups, showed the most misclassified instances with resting. This could be the result of a vague definition of resting in comparison with sleeping, running, and most other activities. An educated guess of how the activities would be ranked from most vague definition to least vague would be: resting, playing, sports, cleaning, cooking, running, smoking, and sleeping. Resting could include naps, sitting behind a computer, reading a book, watching TV, hanging out with friends and family, or going for a short walk. All of these activities could have very different values for the observed variables. This is also true for sports, which is a wide term, and in the case of this dataset, it does not include running. What category should jogging and speed-walking then fall into?

When separated into more specific activities for group M, these activities showed moderate improvement, especially when considering more relevant activities pertaining to exposure to particulate matter. When dividing cooking into two activities, hot and cold, the results show that cooking using a heat source can be identified more easily and produces better results in terms of classification. A similar trend is present for cleaning, though the results are not as clear as for cooking.

On the other hand, sleeping or smoking are quite well-defined activities where there is little room for subjectivity. Even if smoking indoors includes using vaporizers, hookahs, pipes, or other gadgets, the observed variables would still presumably show similar results (elevated levels of PM, sedentary activity in enclosed space, not moving, relatively high heart rate, during the day, etc.), as would sleeping (in a chair, on a bed, on transport, taking a nap, etc.).

### 4.2. Limitations and Future Work

The shallow algorithms used in this research could be replaced by deep learning algorithms that could provide more accurate data. Additional steps could be taken to improve accuracy, e.g., noise removal, scaling, feature extraction, segmentation, and hyperparameter tuning. On the other hand, these approaches would be less understandable to non-experts. It could also limit accessibility in terms of the available software. In participatory-based research, frequently led by non-experts in the field of ML, the selection of algorithms and approaches should be considered based on several factors, apart from accuracy—computation requirements, visualisation options, understandable and explainable architectures/principles, etc. Similarly, more complex approaches used in participatory research could be exclusionary, as they would be more difficult to understand for lay individuals, even more so for individuals with less technical skills and knowledge.

Moreover, more ambiguous or subjectively defined activities should be separated into better-defined activities, as listed above. Although this would impose greater challenges when collecting data for the participants, it could provide more detailed final results and improve the classification. The consequences of these challenges can be seen in Table 1, with fewer recorded instances of the more specific activities in Group M, leading to, in some cases, an unbalanced dataset. A necessary focus would be to evaluate which of these activities are more relevant to the specific study or research and only use the classification models to predict those.

Overall, several additional suggestions and possible improvements are proposed for future research:

- Use of direct movement sensor (accelerometer, gyroscope, magnetometer) data from the SAT.
- Addition of possible other variables to be measured with SAT, e.g., skin temperature and conductivity.
- Utilisation of the data from smartphones (light, movement, location, indoor/outdoor, crowd density, barometer, accelerometer, gyroscope, magnetometer, etc.).
- Fusion of data with government monitoring station data to improve correlations of the measured temperature and humidity.
- Use of static sensor data at home, at the workplace, or in the car to improve or correct measurements made by wearable sensors.
- improvement of an app for logging activity data by providing the participant with (a) a warning when the devices detect a possible change of activity due to changes in parameters and (b) providing suggestions for possible activities ranked from most likely to least likely based on this research.

Half of the challenges listed in Section 1.2 remain unaddressed within the scope of this research, i.e., challenges 2, 3, and 6. While the demonstrated approach in this work does not require detailed personal data (as described in challenge 6), arguably the predictive performance could potentially be improved by including personal characteristics and GPS tracking. Moreover, the inclusion of ambient environmental data do not provide any tangible solutions to increase the quantity of annotated data or reduce sensory data heterogeneity. An argument could be made, to a degree, that collecting data on more variables could require fewer instances of annotated data.

An important improvement to participatory studies would be to reduce the burden of participants filling out time activity diaries while simultaneously reducing the chance of human error. This research shows that machine learning, informed by low-cost personal environmental monitors, can improve the process of recording activity data by reducing or potentially, in the future, completely freeing study participants from recording their activities. Combining the results of this research with environmental stressors measured with portable, low-cost sensors will provide a more detailed picture of exposure and intake dose on an individual scale. Further research is needed to test, validate, and improve these approaches.

As low-cost sensors become more widely used and individuals are able to gain access to more information about their living environment, researchers must provide adequate tools to assess and improve accuracy. A promising step forward would be to reduce the input of individuals and increase the role of machine learning. This research shows that a novel approach to using classification methods with data from low-cost portable environmental and activity sensors can be used to recognise specific activities without direct manual human input.

## Figures and Tables

**Table 1:** Activities and number of instances of each activity in each of the two datasets.

Group M		Group H	
Activity/Task	Nr.	Activity/Task	Nr.
Cleaning.dry.in	438	Cleaning.in	5000
Cleaning.steam.in	416	Cooking.in	5000
Cleaning.wet.in	516	Playing.in	5000
Cooking.cold.in	387	Resting.in	5000
Cooking.hot.in	1923	Running.out	5000
Play.on.feet.in	85	Sleep.in	5000
Play.sedentary.in	469	Smoking.in	5000
Resting.in	5000	Sports.in	5000
Resting.out	225	Sports.out	5000
Running.out	80		
Sleeping.in	5000		
Smoking.in	769		
Sports.in	361		
Sports.out	774		

**Table 2:** Basic statistics for all numeric variables in the dataset.

	Median		Mean		st. dev.		Max		Min		1stQ		3rdQ	
Variable	H	M	H	M	H	M	H	M	H	M	H	M	H	M
PM_1_ [µg/m^3^]	10.0	9.0	18.7	15.4	29.9	26.1	180.0	180.0	0.0	0.0	5.0	4.0	19.0	19.0
PM_2.5_ [µg/m^3^]	13.0	13.0	25.7	21.8	37.5	30.4	180.0	180.0	0.0	0.0	7.0	6.0	28.0	29.0
PM_10_ [µg/m^3^]	15.0	14.0	28.7	24.1	39.1	31.8	180.0	180.0	0.0	0.0	8.0	7.0	31.0	31.0
Temperature [°C]	24.0	23.8	23.6	23.6	3.3	3.2	34.3	34.6	5.9	8.5	22.4	22.4	25.4	25.0
Relative humidity [%]	32.2	39.6	32.8	39.9	8.3	6.9	76.5	67.2	6.7	19.7	27.2	35.4	38.0	44.4
Speed [km/h]	0.6	0.0	1.5	0.1	2.4	0.6	20.0	10.6	0.0	0.0	0.0	0.0	2.0	0.0
Avg. heart rate [bpm]	83.0	69.0	86.2	73.2	22.4	20.0	195.0	177.0	38.0	38.0	70.0	58.0	98.0	85.0
Steps [nr.]	0.0	0.0	16.7	6.1	37.5	20.0	245.0	157.0	0.0	0.0	0.0	0.0	0.0	0.0
M.E.T. [mL O2/kg/min]	0.1	0.1	0.3	0.5	0.5	0.5	15.0	6.1	0.0	0.0	0.1	0.1	0.2	1.0

**Table 3:** Classifiers in WEKA used for this research, with short descriptions.

Classifier	Description
IBk	Instance-based learner [39], otherwise known as the *k*-nearest neighbour (*k*NN) classifier; *k*NN takes the *k* closest examples (typically according to a Euclidean distance) to the given instance in the feature space and counts how many of the *k* belong to each class. The new instance object is classified by plurality vote.
J48	J48 is a Java implementation of the C4.5 decision tree algorithm developed by Ross Quinlan [40]. It can be used for classification and allows a high number of attributes. Deemed as a “machine learning workhorse”, ranked no. 1 in the Top 10 Algorithms in Data Mining [81]. To classify data from a testing set, each sample from the data are propagated through the tree (according to the conditions satisfied by its attribute values). When an example reaches a leaf node, it is assigned the class value of that node.
Random Forest	Constructs a forest of decision trees in a randomized manner. Developed by Leo Breiman [42]. The Random Forest (RF) method is an ensemble learning method for classification that constructs a forest of decision trees in a randomised fashion. Each tree is constructed from a different randomly selected subset of the dataset (bootstrap/sample), with a subset of (randomly chosen) features considered to select a split at each step of tree construction. When the forest is applied to a new instance, each tree votes for one class. The output is the class that gets the most votes from the individual trees.

**Table 4:** Feature merits (importance scores) and ranks for group M.

Group M		
Average Merit	Average Rank	Attribute
0.127 ± 0.001	1 ± 0	Time
0.052 ± 0.001	2 ± 0	Heart rate
0.036 ± 0	3 ± 0	Humidity
0.03 ± 0	4 ± 0	Temperature
0.028 ± 0.001	5 ± 0	PM_10_
0.02 ± 0	6 ± 0	PM_2.5_
0.017 ± 0	7 ± 0	Speed
0.016 ± 0	8 ± 0	Steps
0.014 ± 0	9.2 ± 0.4	PM_1_
0.013 ± 0.001	9.8 ± 0.4	M.E.T.

**Table 5:** Feature merits (importance scores) and ranks for group H.

Group H		
Average Merit	Average Rank	Attribute
0.193 ± 0.001	1 ± 0	Time
0.017 ± 0.001	2.5 ± 0.5	Humidity
0.017 ± 0	2.5 ± 0.5	Heart rate
0.016 ± 0	4	Temperature
0.015 ± 0	5	Steps
0.007 ± 0	6	Speed
0.001 ± 0.001	7	M.E.T.
−0.006 ± 0	8	PM_1_
−0.008 ± 0.001	9	PM_10_
−0.011 ± 0	10	PM_2.5_

**Table 6:** Summary of results for all models for both groups.

Classifier/Metric	CC [%]		Kappa		TP		FP		Precision		Recall		F-Measure		ROC-AUC	
	H	M	H	M	H	M	H	M	H	M	H	M	H	M	H	M
IBk	35.2	34.3	0.3	0.2	0.4	0.3	0.1	0.1	0.4	NA	0.4	0.3	0.4	NA	0.6	0.6
J48	46.5	76.9	0.4	0.7	0.5	0.8	0.1	0.1	0.5	0.8	0.5	0.8	0.5	0.8	0.8	1
RF	52.9	77.2	0.5	0.7	0.5	0.8	0.1	0.1	0.5	0.8	0.5	0.8	0.5	0.8	0.9	1

**Table 7:** Summary for group H, showing TP, FP, precision, recall, F-measure, and ROC-AUC for all classifiers.

	TP			FP			Precision			Recall			F-measure			ROC-AUC		
Class/Classifier	IBk	J48	RF	IBk	J48	RF	IBk	J48	RF	IBk	J48	RF	IBk	J48	RF	IBk	J48	RF
Cleaning.in	0.3	0.4	0.5	0	0.1	0.1	0.5	0.4	0.5	0.3	0.4	0.5	0.4	0.4	0.5	0.6	0.8	0.8
Cooking.in	0.5	0.5	0.4	0.1	0.1	0.1	0.3	0.4	0.5	0.5	0.5	0.4	0.4	0.4	0.4	0.7	0.8	0.8
Playing.in	0.3	0.4	0.4	0.1	0.1	0.1	0.3	0.4	0.5	0.3	0.4	0.4	0.3	0.4	0.5	0.6	0.8	0.8
Resting.in	0.4	0.3	0.3	0.2	0.1	0.1	0.2	0.3	0.3	0.4	0.3	0.3	0.3	0.3	0.3	0.6	0.7	0.7
Running.out	0.3	0.6	0.8	0	0	0	0.6	0.7	0.7	0.3	0.6	0.8	0.4	0.6	0.7	0.7	0.9	1
Sleep.in	0.7	0.8	0.8	0	0	0	0.8	0.7	0.7	0.7	0.8	0.8	0.7	0.8	0.8	0.8	1	1
Smoking.in	0.4	0.3	0.5	0.1	0	0.1	0.3	0.5	0.5	0.4	0.3	0.5	0.3	0.4	0.5	0.6	0.8	0.9
Sports.in	0.2	0.4	0.6	0	0.1	0.1	0.5	0.5	0.6	0.2	0.4	0.6	0.3	0.5	0.6	0.6	0.8	0.9
Sports.out	0.2	0.4	0.5	0	0.1	0.1	0.4	0.4	0.5	0.2	0.4	0.5	0.3	0.4	0.5	0.6	0.8	0.9
Weighted average	0.4	0.5	0.5	0.1	0.1	0.1	0.4	0.5	0.5	0.4	0.5	0.5	0.4	0.5	0.5	0.6	0.8	0.9

**Table 8:** Summary for group M, showing TP, FP, precision, recall, F-measure, and ROC-AUC for all classifiers.

	TP			FP			Precision			Recall			F-Measure			ROC-AUC		
Class	IBk	J48	RF	IBk	J48	RF	IBk	J48	RF	IBk	J48	RF	IBk	J48	RF	IBk	J48	RF
Cleaning.dry.in	0	0.5	0.4	0	0	0	0.1	0.6	0.6	0	0.5	0.4	0	0.6	0.5	0.5	1	1
Cleaning.steam.in	0	0.6	0.5	0	0	0	NA	0.6	0.7	0	0.6	0.5	NA	0.6	0.6	0.5	1	1
Cleaning.wet.in	0	0.4	0.5	0	0	0	NA	0.6	0.5	0	0.4	0.5	NA	0.5	0.5	0.5	0.9	1
Cooking.cold.in	0	0.4	0.4	0	0	0	0.6	0.5	0.4	0	0.4	0.4	0	0.4	0.5	0.5	0.9	1
Cooking.hot.in	0.3	0.8	0.7	0.3	0.1	0.1	0.1	0.6	0.6	0.3	0.8	0.7	0.2	0.7	0.7	0.5	0.9	1
Play.on.feet.in	0	0.6	0.4	0	0	0	NA	0.8	0.9	0	0.6	0.4	NA	0.7	0.6	0.5	0.9	1
Play.sedentary.in	0.5	0.9	0.8	0	0	0	0.4	0.9	0.9	0.5	0.9	0.8	0.4	0.9	0.8	0.7	1	1
Resting.in	0.4	0.7	0.8	0.2	0.1	0.1	0.4	0.8	0.7	0.4	0.7	0.8	0.4	0.8	0.8	0.6	0.9	0.9
Resting.out	0.4	0.4	0.5	0	0	0	0.1	1	0.8	0.4	0.4	0.5	0.2	0.6	0.6	0.7	1	1
Running.out	0.6	0.8	0.9	0.1	0	0	0	0.9	0.9	0.6	0.8	0.9	0.1	0.9	0.9	0.8	1	1
Sleeping.in	0.4	0.9	0.9	0.1	0.1	0.1	0.6	0.8	0.9	0.4	0.9	0.9	0.5	0.9	0.9	0.7	1	1
Smoking.in	0.6	0.7	0.7	0	0	0	0.5	0.9	1	0.6	0.7	0.7	0.5	0.8	0.8	0.7	1	1
Sports.in	0	0.2	0.4	0	0	0	NA	0.8	0.6	0	0.2	0.4	NA	0.4	0.4	0.5	0.9	1
Sports.out	0	0.8	0.9	0	0	0	1	0.9	0.9	0	0.8	0.9	0.1	0.9	0.9	0.5	1	1
Weighted Average	0.3	0.8	0.8	0.1	0.1	0.1	NA	0.8	0.8	0.3	0.8	0.8	NA	0.8	0.8	0.6	1	1

**Table 9:** Instances correctly classified by the J48 and RF models, based on selectively removing all PPM, only PM, and SAT data from the Group M dataset, respectively.

	Correctly Classified Instances [%]			
Classifier	Baseline	no PPM	no PM	no SAT
J48	76.9	60.1	76.2	75.5
RF	77.2	62.5	76.6	77.2

## Data Availability

The datasets generated and/or analysed during the current study are not publicly available due to privacy issues, but are available from the corresponding author on reasonable request.

## Supplementary Materials

The following supporting information can be downloaded at: https://www.mdpi.com/article/10.3390/s23249890/s1, Figure S1: Average values for all variables, per activity, for group H, Figure S2: Average values for all variables, per activity for group M, Table S1: IBk confusion matrix—group H, Table S2: IBk confusion matrix—group M, Table S3: J48 confusion matrix—group H, Table S4: J48 confusion matrix—group M, Table S5: Random Forest confusion matrix—group H, Table S6: Random Forest confusion matrix—group M, Table S7: Feature merits (importance scores) and ranks for group M.
