# Supplementary material for: Empowering Participatory Research in Urban Health: Wearable Biometric and Environmental Sensors for Activity Recognition
Source: Sensors (Basel). 2023 Dec 18;23(24):9890. doi: 10.3390/s23249890 (PMC10747712; doi:10.3390/s23249890)
Supplement: Supplementary file 1 [file sensors-23-09890-s001.zip › sensors-2636081-supplementary.pdf]

Supplementary Material

Empowering Participatory Research in Urban Health: Wearable Biometric and Environmental Sensors for Activity Recognition

Rok Novak[1,2,*], Johanna Amalia Robinson [1,2,3], Tjaša Kanduč [1], Dimosthenis Sarigiannis [4,5,6], Sašo Džeroski [2,7] and David Kocman [1]

1 Department of Environmental Sciences, Jožef Stefan Institute, 1000 Ljubljana, Slovenia; david.kocman@ijs.si (D.K.); tjasa.kanduc@ijs.si (T.K.)

2 Ecotechnologies Programme, Jožef Stefan International Postgraduate School, 1000 Ljubljana, Slovenia; saso.dzeroski@ijs.si (S.D.)

3 Center for research and development, Slovenian Institute for Adult Education, 1000 Ljubljana, Slovenia; johanna.robinson@acs.si (J.A.R)

4 Environmental Engineering Laboratory, Department of Chemical Engineering, Aristotle University of Thessaloniki, Thessaloniki 54124, Greece; denis@eng.auth.gr (D.S.)

5 HERACLES Research Centre on the Exposome and Health, Center for Interdisciplinary Research and Innovation, Thessaloniki 57001, Greece

6 Environmental Health Engineering, Department of Science, Technology and Society, University School of Advanced Study IUSS, 27100 Pavia, Italy

7 Department of Knowledge Technologies, Jožef Stefan Institute, 1000 Ljubljana, Slovenia

* Correspondence: rok.novak@ijs.si (R.N.)

Overview of the dataset: all variables per activity

For a more thorough overview of the dataset, the mean values of all variables for each activity were calculated and plotted in Figure S1 for group H and in Figure S2 for group M.

Running stands out in both groups with the highest values of speed, heart rate, steps, and MET (though the last two are absent for group M due to technical issues with the SAT), and lowest for temperature. sports.OUT and sports.IN also stand out in all these values, while also having low mean PM concentrations. Low PM concentrations for sports are more pronounced in group H, though the values between the IN and OUT activities are reversed between the groups – higher PM for OUT in group H and higher IN for group M. Importantly, sports.IN has also a higher average temperature and relative humidity than sports.OUT.

The highest PM values, by far, are evident for smoking in both groups. This trend is more evident in group M where mean values exceed 80 µg/m^3 and are more than 50 µg/m^3 above the next highest value – cooking. In group H, the difference is between 10 and 20 µg/m^3 (50 µg/m^3 for smoking vs 35 µg/m^3 for cooking). Sports, running, and sleep show the lowest PM values, which is also in line with expectations, as there is less dust resuspension while sleeping and lower concentrations outdoors when performing sports. The slightly lower PM values of sports.IN compared to sports.OUT in group H is more difficult to explain. It could be a consequence of the TAD data logging time resolution in group H, where a person could be performing a sports activity outdoors for only 30 minutes while logging the entire hour as sports.OUT, even if half the hour was spent indoors where PM concentrations were higher. Sleep shows the lowest mean speed, heart rate, number of steps, and MET, all of which is expected. It does not stand out in terms of temperature and humidity.

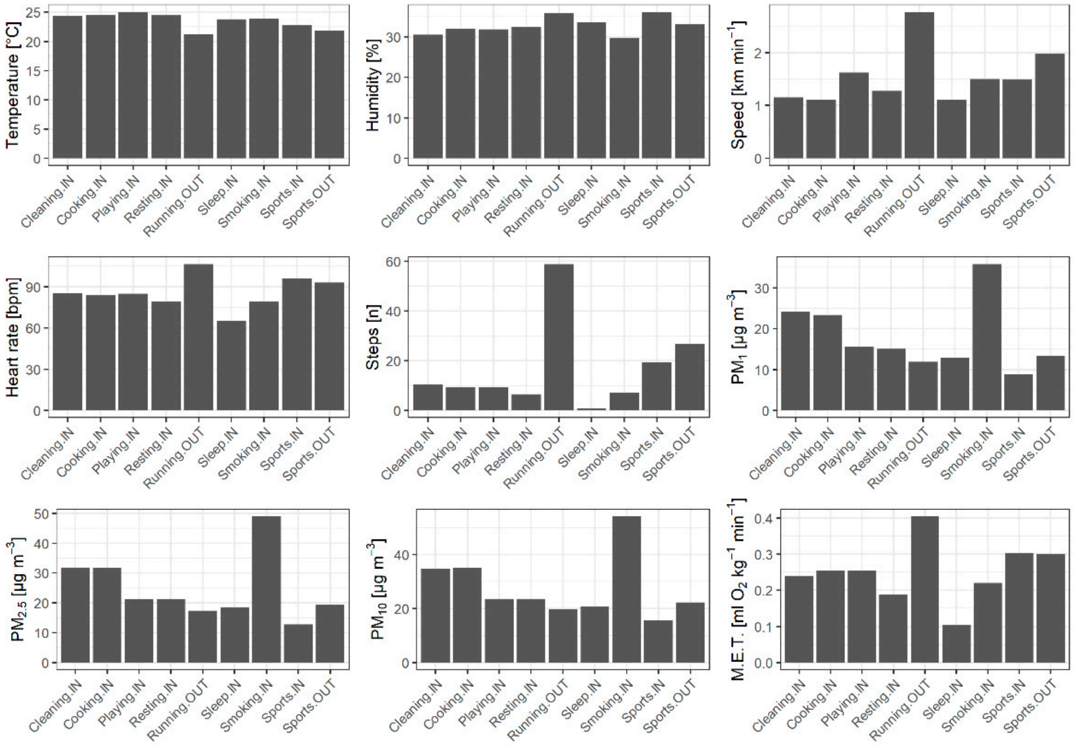

Figure S1 Average values for all variables, per activity, for group H

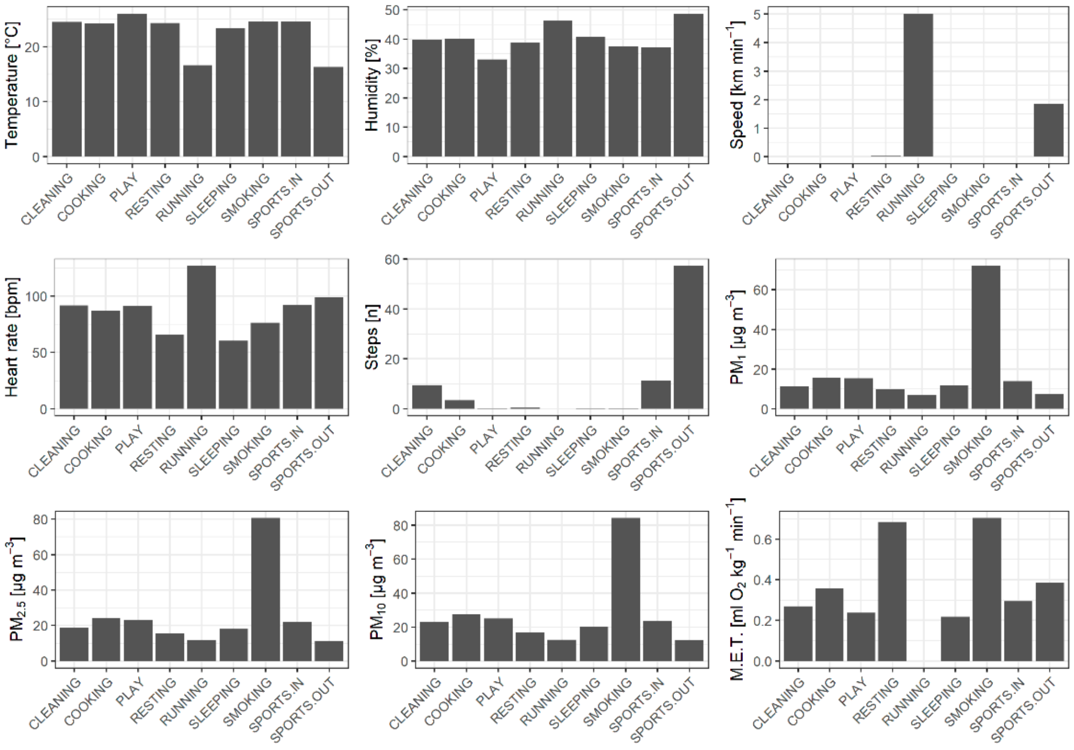

Figure S2 Average values for all variables, per activity for group M

Results: confusion matrices

Results produced in WEKA included confusion matrices for each classifier (IBk, J48, RF) and both groups (group M and group H). Confusion matrices, as described in section 4.6, provide a summary of prediction outcomes on a classification problem and show the total number of correctly classified (diagonal entries) or incorrectly classified instances (all nondiagonal entries) for each class: Each row in the matrix covers all of the

instances that belong to one class (X), and each column covers the instances that are predicted to belong to (classified as) class Y. The confusion matrices for each classifier and each group are shown in Table S1 (IBk, H), Table S2 (IBK, M), Table S3 (J48, H), Table S4 (J48, M), Table S5 (RF, H) and Table S6 (RF, M).

Table S1: IBk confusion matrix - group H

classified as ->	a	b	c	d	e	f	g	h	i
a=cleaning.in	1421	726	616	872	139	115	774	167	170
b=cooking.in	231	2286	297	1033	86	144	634	148	141
c=playing.in	209	621	1430	1353	243	76	699	165	204
d=resting.in	274	664	496	1939	139	261	687	280	260
e=running.out	228	371	545	1299	1680	51	551	99	176
f=sleep.in	100	632	164	330	22	3262	256	80	154
g=smoking.in	128	653	490	1287	122	140	1757	214	209
h=sports.in	102	791	760	1057	97	208	749	1122	114
i=sports.out	167	1226	539	1055	121	59	784	87	962

Table S2: IBk confusion matrix - group M

classified as ->	a	b	c	d	e	f	g	h	i	j	k	l	m	n
a=cleaning.dry.in	3	0	0	2	237	0	67	47	37	18	24	3	0	0
b=cleaning.steam.in	0	0	0	0	137	0	0	57	31	104	21	66	0	0
c=cleaning.wet.in	2	0	0	0	218	0	31	42	44	123	56	0	0	0
d=cooking.cold.in	0	0	0	3	217	0	6	27	18	87	29	0	0	0
e=cooking.hot.in	8	0	0	0	595	0	181	401	155	314	192	77	0	0
f=play.on.feet.in	1	0	0	0	0	0	2	42	10	4	26	0	0	0
g=play.sedentary.in	0	0	0	0	96	0	226	116	0	16	8	7	0	0
h=resting.in	4	0	0	0	1203	0	24	2103	92	608	775	191	0	0
i=resting.out	0	0	0	0	105	0	0	1	84	28	3	4	0	0
j=running.out	1	0	0	0	1	0	11	1	11	50	0	5	0	0
k=sleeping.in	1	0	0	0	883	0	4	1802	14	82	2107	107	0	0
l=smoking.in	0	0	0	0	113	0	12	132	0	68	6	438	0	0
m=sports.in	0	0	0	0	195	0	7	44	5	53	57	0	0	0
n=sports.out	6	0	0	0	251	0	8	95	307	74	1	0	0	32

Table S3: J48 confusion matrix - group H

classified as ->	a	b	c	d	e	f	g	h	i
a=cleaning.in	2166	797	317	502	159	171	210	356	322
b=cooking.in	404	2708	451	354	83	143	142	325	390
c=playing.in	570	782	1761	644	237	78	141	264	523
d=resting.in	495	645	556	1599	175	348	366	307	509
e=running.out	430	342	391	243	2921	98	124	154	297
f=sleep.in	109	60	21	321	39	4189	64	183	14
g=smoking.in	373	599	432	769	233	368	1546	227	453
h=sports.in	249	582	502	429	171	135	210	2120	602
i=sports.out	511	1146	496	191	168	107	103	373	1905

Table S4: J48 confusion matrix - group M

classified as ->	a	b	c	d	e	f	g	h	i	j	k	l	m	n
a=cleaning.dry.in	226	16	14	6	105	1	6	56	0	0	0	0	1	7
b=cleaning.steam.in	6	248	3	4	22	0	0	128	0	0	0	3	1	1
c=cleaning.wet.in	8	44	208	16	121	0	3	97	0	0	5	4	4	6
d=cooking.cold.in	4	24	11	153	134	0	0	55	0	1	1	2	1	1
e=cooking.hot.in	25	11	35	48	1511	1	8	216	2	1	38	3	5	19
f=play.on.feet.in	6	0	3	6	8	51	1	1	0	1	0	0	8	0
g=play.sedentary.in	2	0	2	4	37	1	405	15	0	0	0	1	0	2
h=resting.in	35	30	12	42	254	2	17	3738	0	0	818	47	1	4
i=resting.out	13	0	2	1	66	0	2	30	99	0	0	0	0	12
j=running.out	0	0	0	2	1	0	3	2	0	65	0	2	0	5
k=sleeping.in	1	18	0	8	20	0	21	247	0	0	4684	0	1	0
l=smoking.in	4	5	7	6	96	0	3	102	0	0	2	544	0	0
m=sports.in	5	20	23	2	136	5	6	58	0	0	16	2	87	1
n=sports.out	34	0	22	21	44	0	0	15	1	5	11	0	0	621

Table S5: Random Forest confusion matrix - group H

classified as ->	a	b	c	d	e	f	g	h	i
a=cleaning.in	2329	416	396	431	212	168	329	305	414
b=cooking.in	446	2198	465	351	151	162	323	344	560
c=playing.in	451	404	2208	535	244	85	348	308	417
d=resting.in	509	455	560	1355	251	380	605	355	530
e=running.out	275	123	167	242	3752	99	63	137	142
f=sleep.in	133	66	46	266	41	4201	120	102	25
g=smoking.in	257	340	351	583	249	344	2350	212	314
h=sports.in	220	293	277	452	175	146	213	2802	422
i=sports.out	313	517	344	263	209	114	252	377	2611

Table S6: Random Forest confusion matrix - group M

classified as ->	a	b	c	d	e	f	g	h	i	j	k	l	m	n
a=cleaning.dry.in	192	0	19	20	90	0	0	79	1	0	0	0	15	22
b=cleaning.steam.in	1	201	34	3	17	0	0	151	0	0	0	1	8	0
c=cleaning.wet.in	5	22	267	27	86	0	0	72	2	0	4	2	23	6
d=cooking.cold.in	12	2	18	166	115	0	0	53	0	0	4	2	10	5
e=cooking.hot.in	27	17	63	57	1373	0	0	287	7	0	33	4	18	37
f=play.on.feet.in	2	0	4	5	20	37	1	2	0	0	0	0	10	4
g=play.sedentary.in	1	0	5	4	44	1	361	23	0	1	16	1	4	8
h=resting.in	33	22	28	51	201	0	2	4145	1	3	494	3	9	8
i=resting.out	2	0	0	4	65	0	0	31	102	0	0	0	1	20
j=running.out	0	0	0	0	6	0	0	0	1	71	0	0	0	2
k=sleeping.in	0	10	1	6	2	0	22	484	0	0	4475	0	0	0
l=smoking.in	6	2	6	2	87	0	1	132	0	0	8	516	9	0
m=sports.in	3	0	34	4	98	1	3	64	0	1	17	0	131	5
n=sports.out	13	2	10	26	23	0	0	11	14	0	8	2	0	665
